# Risk factors for trastuzumab-induced cardiotoxicity in HER2-positive breast cancer patients

**DOI:** 10.3389/fonc.2026.1819766

**Published:** 2026-04-22

**Authors:** Hao Zhang, Shenbao Hu, Niya Kong

**Affiliations:** 1 Department of Thyroid and Breast Surgery, Wuhan Third Hospital, Tongren Hospital of Wuhan University, Wuhan, Hubei, China; 2 Department of Thyroid and Breast Surgery, Jingmen Central Hospital affiliated to Jingchu University of Technology, Jingmen, Hubei, China; 3 Department of General Surgery, Jingmen Central Hospital affiliated to Jingchu University of Technology, Jingmen, Hubei, China

**Keywords:** breast cancer, cardiotoxicity, HER2-positive, risk factors, trastuzumab

## Abstract

**Objective:**

To analyze the risk factors for cardiotoxicity induced by trastuzumab in human epidermal growth factor receptor 2 (HER2) -positive breast cancer patients and provide a basis for early clinical intervention.

**Methods:**

A retrospective analysis was conducted on 298 HER2-positive breast cancer patients treated with trastuzumab. Clinical data including age, history of cardiovascular disease, combined chemotherapy regimens, N-terminal pro-brain natriuretic peptide (NT-proBNP) levels, and baseline left ventricular ejection fraction (LVEF) were collected. Multivariate logistic regression was used to identify independent risk factors for cardiotoxicity.

**Results:**

A total of 65 patients (21.8%) developed cardiotoxicity. Multivariate analysis showed that age ≥60 years (OR = 1.97, 95%CI: 1.21–3.22), history of hypertension (OR = 2.10, 95%CI: 1.24–3.56), combined anthracycline therapy (OR = 3.06, 95%CI: 1.67–5.62), baseline NT-proBNP ≥200 pg/ml (OR = 2.34, 95%CI: 1.35–4.05), and baseline LVEF ≤55% (OR = 2.51, 95%CI: 1.42–4.43) were independent risk factors for trastuzumab-induced cardiotoxicity (all P < 0.05).

**Conclusion:**

These findings enable risk stratification before trastuzumab initiation. Future research should validate a predictive model incorporating these factors and assess cardioprotective strategies, thereby translating risk assessment into actionable protocols to optimize cardiac safety without compromising oncologic outcomes.

## 1 Introduction

Breast cancer is a highly heterogeneous malignant tumor with significant differences in histological types and clinical features (1). Selecting individualized treatment plans based on molecular subtypes is key to improving prognosis. Hormone receptor-positive ductal breast cancer is suitable for endocrine therapy. The human epidermal growth factor receptor 2 (HER2) overexpression subtype accounts for approximately 15%–20% of all breast cancers (2). This subtype is highly aggressive and has a poor prognosis but is sensitive to HER2-targeted drugs such as trastuzumab (3).

Trastuzumab is an effective and well-tolerated drug used primarily for the treatment of patients with HER2-positive metastatic breast cancer. It is commonly used as monotherapy or in combination with other chemotherapeutic agents. Compared to chemotherapy alone, the combination of trastuzumab and chemotherapy provides significant clinical benefits to patients by improving disease-free survival and overall survival rates by 33%–52% and 34%–41%, respectively (4).

However, trastuzumab can cause various adverse reactions, including diarrhea, rash, liver injury, neutropenia, and cardiac dysfunction (5). The trastuzumab-induced cardiotoxicity (TIC) is associated with the blockade of HER2 signaling in cardiomyocytes (6). Current research on risk factors for TIC is limited and conclusions are inconsistent. It was previously believed that hypertension, smoking, family history of coronary heart disease, and low baseline left ventricular ejection fraction (LVEF) were the main predictive factors (7, 8). However, recent studies suggest that the cumulative dose of anthracycline drugs (e.g., doxorubicin) is a key risk factor, rather than underlying cardiac disease or concomitant medications (9). Therefore, identifying high-risk patients and implementing early intervention are crucial for improving treatment safety. This study aimed to systematically analyze risk factors associated with TIC to provide evidence for clinical risk prevention and control.

## 2 Materials and methods

### 2.1 Study subjects

This was a single-center, retrospective, observational study conducted at Wuhan Third Hospital, Tongren Hospital of Wuhan University. The subjects were female patients diagnosed with HER2-positive primary invasive breast cancer by core needle biopsy pathology at the Department of Thyroid and Breast Surgery, Wuhan Third Hospital, Tongren Hospital of Wuhan University, between January 2018 and December 2022. Electronic medical records of HER2-positive breast cancer patients who received trastuzumab-targeted therapy were retrieved through our hospital’s record system. Follow-up was conducted through outpatient review records, inpatient medical records, and telephone interviews. The follow-up cutoff date was December 2024.All patients had signed informed consent, and this study was approved by the hospital’s medical ethics committee.

Inclusion criteria: ① Pathologically confirmed HER2-positive invasive breast cancer (IHC 3+ or FISH positive); ② Received ≥4 cycles of trastuzumab treatment (monotherapy or combined with chemotherapy); ③ Baseline LVEF ≥50% and no clinical symptoms of heart failure. Exclusion criteria: ① Previous use of other HER2 inhibitors (e.g., pertuzumab, T-DM1); ② Acute coronary syndrome or stroke within 12 months before treatment; ③ Severe renal insufficiency (glomerular filtration rate (GFR) <30 ml/min/1.73m²); ④ Patients with baseline LVEF <50% (i.e., with overt systolic dysfunction) were strictly excluded. ⑤Missing key variables in clinical data (e.g., LVEF, drug dosage). ⑥Patients with chronic ischemic heart disease, heart failure, or valvular heart disease. Baseline assessments, including LVEF and NT−proBNP, were performed after completion of any neoadjuvant or adjuvant chemotherapy and immediately before the first administration of trastuzumab.

### 2.2 Research methods

Trastuzumab was used according to the drug instructions. Chemotherapy regimens and endocrine therapy complied with Chinese Society of Clinical Oncology (CSCO) guidelines, with specific drug doses calculated based on body surface area. Radiotherapy was administered as indicated, with dose and fractionation determined based on the actual situation. Cardiotoxicity was defined according to the 2016 ESC Position Paper on cancer treatments and cardiovascular toxicity (10).

Diagnostic criteria for cardiotoxicity: (1) Cardiomyopathy with reduced LVEF, manifested as decreased global function or significant reduction in septal motion; (2) Symptoms associated with congestive heart failure (CHF); (3) Signs related to congestive heart failure, such as third heart sound gallop, tachycardia, or both; (4) Absolute LVEF decrease of at least 5% to <55% from baseline, accompanied by CHF symptoms, OR absolute LVEF decrease of at least 10% to <55% without CHF symptoms or signs (10). Enrolled patients were divided into a cardiotoxicity group and a non-cardiotoxicity group based on the presence or absence of cardiotoxicity. Clinical data recorded included age, body mass index (BMI), tumor stage, tumor location, tumor histology, hypertension, hyperlipidemia, diabetes, thyroid function, chemotherapy, radiotherapy, endocrine therapy, estrogen receptor (ER) status, progesterone receptor (PR) status, baseline LVEF, baseline N-terminal pro-brain natriuretic peptide (NT-proBNP), anthracycline sequential therapy, etc.

### 2.3 Statistical methods

Data were statistically analyzed using SPSS 20.0. Count data were expressed as percentages. Univariate analysis was performed using the χ² test or Fisher’s exact test. Based on the results, variables with statistical significance were used as independent variables, and the occurrence of cardiotoxicity was used as the dependent variable for logistic regression analysis. A P-value <0.05 was considered statistically significant.

## 3 Results

### 3.1 Profile of breast cancer patients treated with trastuzumab

A total of 332 patients was identified as receiving trastuzumab (Herceptin^®^, F. Hoffmann-La Roche Ltd). Thirty-four patients were excluded from the study sample, including 6 patients with a history of cardiomyopathy and 28 patients without LVEF assessment before or after trastuzumab administration. The median age of the enrolled patients was 52 years (range 38–76 years). The average age of eligible patients was 52.7 ± 12.2 years. Most patients were overweight or obese (37.0% and 45.9%, respectively). All patients received trastuzumab treatment for invasive ductal carcinoma. The majority of patients (91.1%) had unilateral tumors and underwent either total mastectomy (51.4%) or lumpectomy (17.8%). The median follow-up duration was 26 months (interquartile range, 18–34 months). **Table 1** lists the basic profile of breast cancer patients treated with trastuzumab included in this study.

**Table 1:** Profile of breast cancer patients treated with trastuzumab (n = 298).

Variable	Value
Age (years)	
Mean ± SD	52.7 ± 12.2
Median (Range)	52 (38-76)
BMI, n (%)	
<18.5 (Underweight)	2 (0.7%)
18.5–24.9 (Healthy weight)	49 (16.4%)
25–29.9 (Overweight)	110 (37.0%)
>30 (Obese)	137 (45.9%)
Menopausal status, n (%)	
Premenopausal	133 (44.6%)
Postmenopausal	165 (55.4%)
Allergy history, n (%)	
No	271 (91.0%)
Yes	27 (9.0%)
Hypertension, n (%)	
No	198 (66.4%)
Yes	100 (33.6%)
Hyperlipidemia, n (%)	
No	237 (79.5%)
Yes	61 (20.5%)
Diabetes, n (%)	
No	214 (71.8%)
Yes	84 (28.2%)
Thyroid dysfunction, n (%)	
Normal	261 (87.6%)
Hypothyroidism	18 (6.0%)
Hyperthyroidism	19 (6.4%)
Histological cancer type, n (%)	
Ductal carcinoma	298 (100.0%)
Lobular carcinoma	0 (0.0%)
TNM stage, n (%)	
Stage I	16 (5.4%)
Stage II	106 (35.6%)
Stage III	92 (30.9%)
Stage IV	84 (28.1%)
Tumor site, n (%)	
Unilateral	271 (91.0%)
Bilateral	27 (9.0%)
Surgery, n (%)	
No	92 (30.9%)
Yes: Total mastectomy	153 (51.3%)
Lumpectomy	53 (17.8%)
Baseline LVEF (%)	
50%–55%	157 (52.7%)
≥55%	141 (47.3%)
Baseline NT-proBNP	
< 200 pg/ml	182 (61.1%)
≥ 200 pg/ml	116 (38.9%)
Estrogen receptor, n (%)	
Positive	167 (56.0%)
Negative	131 (44.0%)
Progesterone receptor, n (%)	
Positive	141 (47.3%)
Negative	157 (52.7%)
HER2 expression, n (%)	
2+	41 (13.8%)
3+	257 (86.2%)
Trastuzumab, n (%)	
Adjuvant/Neoadjuvant	210 (70.5%)
Palliative	88 (29.5%)
Radiotherapy, n (%)	
No	163 (54.7%)
Yes	135 (45.3%)
Endocrine therapy, n (%)	
No	149 (50.0%)
Yes: Tamoxifen	73 (24.5%)
Anastrozole/Letrozole	94 (31.5%)
Both	18 (6.0%)
Anthracycline, n (%)	
No	200 (67.1%)
Yes: Doxorubicin	31 (10.4%)
Epirubicin	67 (22.5%)
Taxane, n (%)	
No	122 (40.9%)
Yes: Docetaxel	106 (35.6%)
Paclitaxel	70 (23.5%)
Concurrent Anthracycline and Taxane	
No	208 (69.8%)
Yes	90 (30.2%)

LVEF, left ventricular ejection fraction; NT-proBNP, N-terminal pro-brain natriuretic peptide; BMI, body mass index; TNM,Tumor-Node-Metastasis.

### 3.2 Analysis of factors associated with cardiotoxicity in breast cancer patients

Among the 298 patients, a comparative study of various factors between the cardiotoxicity and non-cardiotoxicity groups revealed significant differences between the two groups in age, hypertension, baseline LVEF (%), baseline NT-proBNP (pg/ml), and anthracycline sequential therapy, as detailed in **Table 2**.

**Table 2:** Analysis of factors associated with cardiotoxicity in breast cancer patients.

Variable	Cardiotoxicity		χ²	P-value
	Yes (n=65)	None (n=233)		
Age (years)			4.013	0.031
<60	27 (41.5%)	100 (42.9%)		
≥60	38 (58.5%)	133 (57.1%)		
BMI, n (%)			1.147	0.187
Underweight/Healthy	14 (21.5%)	37 (15.9%)		
Overweight/Obese	51 (78.5%)	196 (84.1%)		
Menopausal status, n (%)			0.001	0.556
Premenopausal	29 (44.6%)	104 (44.6%)		
Postmenopausal	36 (55.4%)	129 (55.4%)		
Hypertension, n (%)			9.0797	0.001
No	39 (60.0%)	159 (68.2%)		
Yes	26 (40.0%)	74 (31.8%)		
Hyperlipidemia, n (%)			0.058	0.465
No	51 (78.5%)	186 (79.8%)		
Yes	14 (21.5%)	47 (20.2%)		
Diabetes, n (%)			2.753	0.067
No	39 (60.0%)	175 (75.1%)		
Yes	26 (40.0%)	58 (24.9%)		
Thyroid dysfunction, n (%)			0.001	0.584
Normal	57 (87.7%)	204 (87.6%)		
Abnormal	8 (12.3%)	29 (12.4%)		
TNM stage, n (%)			1.525	0.139
Stage I/II	16 (24.6%)	106 (45.5%)		
Stage III/IV	49 (75.4%)	127 (54.5%)		
Tumor site, n (%)			2.794	0.076
Unilateral	53 (81.5%)	218 (93.6%)		
Bilateral	12 (18.5%)	15 (6.4%)		
Surgery, n (%)			1.426	0.149
No	24 (36.9%)	68 (29.2%)		
Yes	41 (63.1%)	165 (70.8%)		
Baseline LVEF (%)			6.746	0.007
50%—55%	25 (38.5%)	132 (56.7%)		
≥55%	40 (61.5%)	101 (43.3%)		
Baseline NT-proBNP			9.472	0.002
< 200 pg/ml	29 (44.6%)	153 (65.7%)		
≥ 200 pg/ml	36 (55.4%)	80 (34.3%)		
Estrogen receptor, n (%)			0.938	0.204
Positive	33 (50.8%)	134 (57.5%)		
Negative	32 (49.2%)	99 (42.5%)		
Progesterone receptor, n (%)			1.785	0.116
Positive	26 (40.0%)	115 (49.4%)		
Negative	39 (60.0%)	118 (50.6%)		
HER2 expression, n (%)			0.114	0.435
2+	10 (15.4%)	31 (13.3%)		
3+	55 (84.6%)	202 (86.7%)		
Trastuzumab, n (%)			0.296	0.345
Adjuvant/Neoadjuvant	39 (60.0%)	171 (73.4%)		
Palliative	26 (40.0%)	62 (26.6%)		
Radiotherapy, n (%)			0.024	0.493
No	35 (53.8%)	128 (54.9%)		
Yes	30 (46.2%)	105 (45.1%)		
Endocrine therapy, n (%)			0.177	0.390
No	31 (47.7%)	118 (50.6%)		
Yes	34 (52.3%)	115 (49.4%)		
Anthracycline, n (%)			15.143	<0.001
No	45 (69.2%)	155 (66.5%)		
Yes	20 (30.8%)	78 (33.5%)		
Taxane, n (%)			0.089	0.440
No	24 (36.9%)	98 (42.1%)		
Yes	41 (63.1%)	135 (57.9%)		

LVEF, left ventricular ejection fraction; NT-proBNP, N-terminal pro-brain natriuretic peptide; BMI, body mass index; TNM,Tumor-Node-Metastasis.

Continuous variables were compared using Student’s t−test or Mann−Whitney U test as appropriate; categorical variables were compared using χ² test or Fisher’s exact test.

### 3.3 Univariate analysis of risk factors for trastuzumab-induced cardiotoxicity

Univariate analysis of risk factors in 298 patients from the cardiotoxicity group identified age, history of hypertension, anthracycline sequential therapy, baseline NT-proBNP ≥200 pg/ml, and baseline LVEF 50%—55% as important risk factors, as detailed in **Table 3**.

**Table 3:** Univariate analysis of risk factors for trastuzumab-induced cardiotoxicity.

Variable	β	S.E.	Wald	df	Exp (β)	95% C.I.	Sig.
Age ≥60 years	0.76	0.32	5.64	1	2.14	1.15–4.01	**0.018**
Hypertension	0.85	0.34	6.25	1	2.34	1.20–4.55	**0.013**
Diabetes	0.31	0.29	1.14	1	1.36	0.77–2.41	0.285
Hyperlipidemia	0.12	0.38	0.1	1	1.13	0.54–2.37	0.752
Baseline LVEF 50%—55%	0.92	0.33	7.78	1	2.51	1.31–4.80	**0.005**
Baseline NT-proBNP ≥200 pg/ml	0.88	0.31	8.05	1	2.41	1.31–4.43	**0.004**
Anthracycline use	1.15	0.35	10.79	1	3.16	1.59–6.27	**0.001**
Taxane use	0.08	0.28	0.08	1	1.08	0.62–1.88	0.781
Radiotherapy	0.07	0.27	0.07	1	1.07	0.63–1.82	0.796

LVEF, left ventricular ejection fraction; NT-proBNP, N-terminal pro-brain natriuretic peptide. Continuous variables were compared using Student’s t−test or Mann−Whitney U test as appropriate; categorical variables were compared using χ² test or Fisher’s exact test. Multivariate logistic regression included variables with P < 0.01 in univariate analysis.

Bold values indicate statistical significance.

### 3.4 Multivariate logistic regression analysis of risk factors for trastuzumab-induced cardiotoxicity

Multivariate logistic regression analysis of risk factors in 298 patients from the cardiotoxicity group identified age, history of hypertension, anthracycline sequential therapy, baseline NT-proBNP ≥200 pg/ml, and baseline LVEF 50%—55% as important risk factors, as detailed in **Table 4**.

**Table 4:** Multivariate logistic regression analysis of risk factors for trastuzumab-induced cardiotoxicity.

Variable	β	S.E.	Wald	df	Exp(β)	95% C.I.	Sig.
Age (per 10-year increase)	0.68	0.25	7.41	1	1.97	1.21–3.22	0.006
Hypertension (Yes vs No)	0.74	0.27	7.51	1	2.1	1.24–3.56	0.005
Baseline LVEF 50%—55%	0.92	0.29	10.05	1	2.51	1.42–4.43	0.001
Baseline NT-proBNP ≥200 pg/ml	0.85	0.28	9.21	1	2.34	1.35–4.05	0.002
Anthracycline sequential therapy (Yes vs No)	1.12	0.31	13.04	1	3.06	1.67–5.62	<0.001

LVEF, left ventricular ejection fraction; NT-proBNP, N-terminal pro-brain natriuretic peptide. Continuous variables were compared using Student’s t−test or Mann−Whitney U test as appropriate; categorical variables were compared using χ² test or Fisher’s exact test. Multivariate logistic regression included variables with P < 0.01 in univariate analysis.

## 4 Discussion

Cardiotoxicity is a known adverse reaction to various anticancer drugs including anthracyclines and trastuzumab. Most patients did not experience significant LVEF decline or heart failure symptoms. Literature reports indicate that the incidence of TIC is 2%–7% when trastuzumab is used as monotherapy and can be as high as 28% when combined with anthracyclines and taxanes (11). TIC mostly manifests as asymptomatic LVEF decline and rarely leads to severe heart failure (12), and is reversible in most patients after drug discontinuation and initiation of heart failure treatment (13).

While the use of trastuzumab significantly improves survival outcomes in HER2-positive breast cancer patients, TIC has become an issue that cannot be ignored in clinical practice. Through a retrospective analysis of clinical data from 298 patients, this study found a TIC incidence of 21.8% and identified multiple independent risk factors, including advanced age (≥60 years), history of cardiovascular disease, combined anthracycline therapy, baseline NT-proBNP ≥200 pg/ml, and baseline LVEF ≤55%. These results are consistent with several domestic and international studies (14–16), further confirming the multifactorial interaction mechanism of TIC.

Currently, domestic and international research on TIC mostly focuses on single factors or small sample analyses, lacking systematic multifactorial comprehensive evaluation. Through multivariate logistic regression analysis, this study not only verified known risk factors such as age, underlying cardiovascular disease, and anthracycline use but also emphasized the important role of baseline NT-proBNP and LVEF in predicting TIC. This aligns with the European Cardio-Oncology guidelines recommending monitoring cardiac function markers at baseline and during treatment (17), further supporting the clinical value of NT-proBNP as an early biomarker for cardiac function injury (18, 19).

Compared with similar foreign studies, the prevalence of hypertension and diabetes in the study population was higher, suggesting that TIC risk may be further elevated in populations with comorbid metabolic diseases (20, 21).

The significance of this study lies in providing a practical tool for clinically identifying high-risk populations for TIC, emphasizing the need for a comprehensive assessment of patients’ cardiovascular status before initiating trastuzumab treatment, especially in elderly patients, those with a history of cardiovascular and cerebrovascular diseases, those planned for combined anthracycline therapy, and those with reduced baseline cardiac function or elevated NT-proBNP. For such patients, it is recommended to strengthen cardiac function monitoring during treatment and implement early intervention when necessary to reduce the risk of TIC.

Based on the findings of this study, it is recommended that before initiating trastuzumab, all HER2-positive breast cancer patients undergo a comprehensive baseline cardiac assessment including LVEF and NT-proBNP measurement to identify high-risk individuals—specifically those aged ≥60 years, with a history of hypertension, planned anthracycline therapy, baseline NT-proBNP ≥200 pg/ml, or baseline LVEF ≤55%. For these high-risk patients, regular cardiac monitoring should be performed every three cycles or more frequently as needed, and prophylactic use of cardioprotective agents such as beta-blockers or ACE inhibitors may be considered in consultation with a cardiologist. Additionally, optimizing the sequencing and dosing of anthracyclines—such as minimizing cumulative doses or using alternative regimens when feasible—can further reduce the risk of cardiotoxicity. Implementing these strategies will help balance the oncologic benefits of trastuzumab with cardiac safety, enabling early intervention and ultimately improving both treatment outcomes and patient quality of life.

Several limitations of this study should be acknowledged. First, its retrospective, single−center design inherently limits generalizability and introduces potential selection bias. Second, although we excluded patients with baseline LVEF <50%, a substantial proportion (52.7%) had baseline LVEF in the range of 50%–55%, which may represent a population with subclinical cardiac vulnerability. Third, we did not collect data on cumulative anthracycline dose or detailed cardiovascular medication use, which could have influenced the risk estimates. Fourth, the follow−up duration varied among patients, and while the median follow−up was 26 months, longer follow−up might capture later−onset cardiotoxicity events. Finally, the absence of central echocardiographic core−lab reading may have introduced variability in LVEF measurements. Although this decision was made pragmatically to reflect real-world clinical scenarios, we performed multivariate regression analyses with and without adjustment for potential confounders to assess the stability of the risk estimates.

TIC is a relatively common adverse event in the majority of HER2-positive breast cancer patients receiving trastuzumab therapy, even occurring after drug withdrawal. This study confirms the importance of continuous screening for any impending cardiotoxicity in breast cancer patients during the treatment period and even after completing chemotherapy. Identifying cardiotoxicity-related factors and early recognition of breast cancer patients at high risk for TIC are crucial for avoiding such harmful adverse reactions and initiating heart failure treatment when needed. Advanced age, underlying cardiovascular disease, combined anthracycline therapy, baseline NT-proBNP ≥200 pg/ml, and baseline LVEF ≤ 55% are independent risk factors for trastuzumab treatment-related cardiotoxicity in HER2-positive breast cancer patients. Clinically, individualized monitoring and management strategies should be developed for the aforementioned high-risk populations to achieve a balance between antitumor therapy and cardiac safety.

## Data Availability

The original contributions presented in the study are included in the article/supplementary material. Further inquiries can be directed to the corresponding author.
